# Dissociated Role of D-Serine in Extinction During Consolidation vs. Reconsolidation of Context Conditioned Fear

**DOI:** 10.3389/fnmol.2018.00161

**Published:** 2018-05-22

**Authors:** Ran Inoue, Gourango Talukdar, Keizo Takao, Tsuyoshi Miyakawa, Hisashi Mori

**Affiliations:** ^1^Department of Molecular Neuroscience, Graduate School of Medicine and Pharmaceutical Sciences, University of Toyama, Toyama, Japan; ^2^Life Science Research Center, University of Toyama, Toyama, Japan; ^3^Section of Behavior Patterns, Center for Genetic Analysis of Behavior, National Institute for Physiological Sciences, Aichi, Japan; ^4^Genetic Engineering and Functional Genomics Group, Frontier Technology Center, Graduate School of Medicine, Kyoto University, Kyoto, Japan; ^5^Division of Systems Medical Science, Institute for Comprehensive Medical Science, Fujita Health University, Toyoake, Japan

**Keywords:** D-serine, serine racemase, contextual fear, extinction, AMPA receptor

## Abstract

Extinction-based exposure therapy is widely used for the treatment of anxiety disorders, such as post-traumatic stress disorder (PTSD). D-serine, an endogenous co-agonist at the glycine-binding site of the *N*-methyl-D-aspartate-type glutamate receptor (NMDAR), has been shown to be involved in extinction of fear memory. Recent findings suggest that the length of time between the initial learning and an extinction session is a determinant of neural mechanism involved in fear extinction. However, how D-serine is involved in extinction of fear memory at different timings remains unclear. In the present study, we investigated the role of D-serine in immediate, delayed and post-retrieval extinction (P-RE) of contextual fear memory using wild-type (WT) and serine racemase (SRR) knockout (KO) mice that exhibit 90% reduction in D-serine content in the hippocampus. We found that SRR disruption impairs P-RE, facilitates immediate extinction (IE), but has no effect on delayed extinction (DE) of contextual fear memories. The impaired P-RE of contextual fear memory in SRRKO mice was associated with increased expression of the GluA1 subunit of the α-amino-3-hydroxy-5-methyl-4-isoxazolepropionic acid-type glutamate receptor (AMPAR) in the hippocampal synaptic membrane fraction after P-RE, and this increase of AMPAR and impaired P-RE were rescued by the administration of D-serine to SRRKO mice. Our findings suggest that D-serine is differentially involved in the regulation of contextual fear extinction depending on the timing of behavioral intervention, and that combining D-serine or other drugs, enhancing the NMDAR function, with P-RE may achieve optimal outcomes for the treatment of PTSD.

## Introduction

The mammalian brain contains high levels of D-serine, which acts as a co-agonist at the glycine-binding site of the *N*-methyl D-aspartate-type glutamate receptor (NMDAR; Hashimoto et al., 1992). The synthesis of D-serine from L-serine is catalyzed by serine racemase (SRR; Wolosker et al., 1999). Studies using SRR knockout (KO) mice have demonstrated that the majority of D-serine is produced by neuronal SRR and that an appropriate level of D-serine is necessary for NMDAR-mediated neurotransmission and long-term potentiation of synaptic transmission in the hippocampus (Inoue et al., 2008; Basu et al., 2009; Balu et al., 2013).

Long-lasting memories of dangerous events are essential for an individual’s survival, but are also implicated in the pathogenesis of anxiety disorders, post-traumatic stress disorder (PTSD) in particular, when they cause excessive fear and anxiety. Extinction-based exposure therapy has been used for the treatment of PTSD, which extinguishes or suppresses the fear response by repeatedly exposing the subjects to fear-inducing stimuli (Roberts et al., 2016). However, the efficacy of the therapy varies across reports (Craske et al., 2008). Thus, understanding the molecular mechanisms underlying fear extinction is of tremendous clinical relevance. Recent findings using rodents have indicated that the length of time between the initial learning and extinction session is a determinant of neural mechanisms for extinguishing an original memory (Rescorla, 2004; Myers et al., 2006; Herry et al., 2010; Maren, 2011; Clem and Schiller, 2016). An extinction training performed 24 h after conditioning, also called delayed extinction (DE), involves the formation of new, inhibitory learning that merely suppresses the original learning (Rescorla and Heth, 1975; Bouton and Bolles, 1979; Robbins, 1990). Extinction training conducted at the time of fear memory reconsolidation (lasts up to 6 h after retrieval) destabilizes the original fear memory trace and recruits erasure-like mechanisms (Monfils et al., 2009; Schiller et al., 2010; Rao-Ruiz et al., 2011). However, there exists inconsistency on the effect of post-retrieval extinction (P-RE) training as it failed to be replicated in other works (Golkar et al., 2012; Almeida-Corrêa and Amaral, 2014). Regarding to immediate extinction (IE) training that is initiated soon after acquisition, prevalent evidence coming from studies (Maren and Chang, 2006; Schiller et al., 2008; Woods and Bouton, 2008; Archbold et al., 2010), except one (Myers et al., 2006), have revealed null effect in reduction of fear expression. Recently, it was found that the IE deficit can be rescued by pharmacological approaches (Hollis et al., 2016; Giustino et al., 2017). Furthermore, in view of the fact that most human studies employ IE training for observing long-term extinction, the effect of IE approach is worthy of being further explored (Lonsdorf et al., 2017).

Like many other forms of learning in mammals, extinction is also dependent on NMDAR function and activation of the NMDAR glycine-binding site has been shown to facilitate extinction of a fear memory. Preclinical and clinical studies suggest that fear extinction can be facilitated with delivery of D-cycloserine, a partial agonist at the glycine-binding site of the NMDAR (Walker et al., 2002; Mao et al., 2006; Mataix-Cols et al., 2017). D-Serine has been demonstrated to enhance the extinction of fear memory in studies using auditory-cued fear conditioning and inhibitory avoidance tasks in rodents (Matsuda et al., 2010; Bai et al., 2014). Moreover, in a study by Labrie et al. (2009) in mice, genetic inactivation of D-amino acid oxidase, a catabolic enzyme of D-serine, enhanced contextual fear extinction through increasing the level of endogenous D-serine level. However, all of these studies focus on DE, and whether a change in D-serine content would influence immediate or P-RE of fear memory remains unknown.

In the present study, we investigated the role of D-serine in immediate, delayed and P-RE of contextual fear memory using wild-type (WT) and SRRKO mice that exhibit a 90% reduction in D-serine content in the hippocampus and cerebral cortex (Inoue et al., 2008). Given that modulation of α-amino-3-hydroxy-5-methyl-4-isoxazolepropionic acid-type glutamate receptor (AMPAR) endocytosis and the resulting synaptic depotentiation are indispensable molecular mechanisms for the behavioral effect of extinction (Dalton et al., 2008; Rao-Ruiz et al., 2011), we further examined the expression levels of GluA1 and GluA2 subunit of AMPAR in the hippocampal synaptic membrane fraction after immediate and P-RE training.

## Materials and Methods

### Mice

WT and SRRKO mice with a pure C57BL/6N genetic background was reported previously (Miya et al., 2008). For the open field and elevated plus maze tests, WT and SRRKO mice were generated by *in vitro* fertilization and transferring fertilized WT and SRRKO embryos to the ovary of pseudopregnant ICR mice. Male WT and SRRKO mice at the age of 3 months were used for the experiments. Animal care and experimental protocols were approved by the Animal Experiment Committee of the University of Toyama (Authorization No. 2013 MED-66), Institutional Animal Care and Use Committee of Graduate School of Medicine of Kyoto University (Authorization No. MedKyo 08165, MedKyo 09539), and National Institute for Physiological Sciences (Authorization No. 09A207). The mice were kept in a temperature- and humidity-controlled room under a 12 h light/dark cycle (lights on at 7:00 AM) and had *ad libitum* access to food and water. Raw data of the behavioral test conducted in Kyoto University and National Institute for Physiological Sciences are open on a public database “Mouse Phenotype Database^1^.”

### Contextual Fear Conditioning and Extinction

Mice were handled daily for 1 min for 1 week before behavioral experiments. Contextual fear conditioning was carried out in a small conditioning chamber (10 × 10 × 10 cm, with a floor made of 14 stainless steel rods) surrounded by a sound-attenuating chest (CL-M3, O’Hara and Co., Tokyo, Japan). Mice were placed in the conditioning chamber and two electrical foot shocks (0.5 mA, 1 s) were delivered 59 and 119 s after entry to the chamber. One minute after the last foot shock, the mice were returned to their home cages. Twenty-four or 48 h later, mice were re-exposed in the conditioned chamber for 5 min to measure the contextual fear memory.

For extinction training, mice were re-exposed to the conditioned chamber for 30 min without receiving a foot shock again. IE and DE was conducted 15 min and 24 h after fear conditioning, respectively. For P-RE, mice were subjected to a 3-min retrieval session 24 h after fear conditioning, and 2 h later, the extinction training was conducted. The recall test of extinction memory was conducted 24 h after extinction training. Freezing responses were analyzed with ImageFZ (Shoji et al., 2014), which is a software based on the NIH Image program. For experiments of fear conditioning and recall test, the capture rate of images was set 2 frame/sec. For the experiment of extinction training, owing to limitation of our software (analyzing 3200 frames), the capture rate of images was set at 1 frame/s.

### Open Field Test

Locomotor activity was measured in an open field apparatus (40 × 40 × 30 cm; Accuscan Instruments, Columbus, OH, USA) as previously described (Ihara et al., 2007). The lighting in the room was set at 100 lux. Each mouse was placed in the center of an open field and the total distance traveled and the time spent in the center (20 × 20 cm) of the open field area were recorded using VersaMax system (Accuscan Instruments) for 120 min. The open field area was cleaned with 70% ethanol before each trial.

### Elevated Plus Maze Test

The elevated plus-maze test was conducted as previously described (Komada et al., 2008). The elevated plus maze (O’Hara and Co., Tokyo, Japan) consisted of two open arms (25 × 5 cm) and two closed arms of same size, with 15 cm high transparent walls. The arms and central square were elevated to a height of 55 cm above the floor. Each mouse was placed in the central square of the maze (5 × 5 cm), facing one of the closed arms. The lighting in the room was set at 100 lux. Mouse behavior was recorded during a 10 min test. The number of entries into, and the time spent on open and enclosed arms were recorded. The arms and central square were cleaned with 70% ethanol before each trial.

### Synaptic Membrane Preparation

WT and SRRKO mice were deeply anesthetized by intraperitoneal injection of pentobarbital sodium (100 mg/kg body weight) at the desired time points and perfused transcardially with ice-cold phosphate-buffered saline (PBS, pH 7.4). Hippocampi were dissected from fresh brains and frozen in liquid N_2_. Samples were stored at −80°C until use. Synaptic membrane fractions were prepared as previously described but with slight modification (Huttner et al., 1983). The hippocampi were homogenized in ice-cold buffered sucrose (0.32 M sucrose and 4 mM HEPES-NaOH, pH 7.4) and then centrifuged at 800× *g* for 10 min at 4°C. The supernatants were centrifuged at 16,000× *g* for 15 min at 4°C. The pellet was resuspended in 2 ml of 2 M sucrose and loaded on top of a sucrose gradient consisting of 0.85 M and 1.2 M sucrose. After centrifugation at 100,000× *g* for 2 h at 4°C, the synaptic membrane fraction at the 0.85/1.2 M sucrose interface was collected. The synaptic membrane fraction was centrifuged at 16,000× *g* for 15 min at 4°C and the pellet was resuspended in ice-cold mammalian protein extraction reagent (Pierce, Rockford, IL, USA). The protein concentration was determined using a BCA Protein Assay kit (Pierce).

### Western Blot Analysis

Western blot analysis was performed as previously described (Inoue et al., 2014). Protein extracts (3 μg) were subjected to SDS-PAGE and separated proteins were transferred onto polyvinylidene difluoride membranes. After blocking with a solution containing 5% skim milk in PBS, the membranes were incubated with rabbit anti-GluA1 (1:2000, catalog #GluR1C-Rb-Af692, Frontier Institute, Hokkaido, Japan) or anti-GluA2 (1:2000, catalog #GluR2C-Rb-Af1050, Frontier Institute) polyclonal antibodies overnight at 4°C, then with an appropriate HRP-conjugated secondary antibody for 1 h at room temperature. Protein bands were detected using an ECL chemiluminescence detection system and an Image Quant LAS 4000 Mini (GE Healthcare, Uppsala, Sweden). For reprobing, the membranes were incubated with stripping buffer (62.5 mM Tris-HCl, 2% SDS, and 100 mM 2-mercaptoethanol) for 30 min at 50°C. The membranes were incubated with rabbit anti-transferrin receptor antibody (1:1000, catalog #ab84036, Abcam, Bristol, UK) and then processed as described above. The signal of the protein bands was quantified using ImageJ 1.46r software.

### D-Serine Treatment

D-Serine (Wako, Tokyo, Japan) was dissolved in saline at 0.27 g/ml and administered to the mice by intraperitoneal injection at 2.7 g/kg body weight. The dose of D-serine (2.7 g/kg) was chosen based on the results of previous behavioral studies (Kanahara et al., 2008; Matsuda et al., 2010).

### Statistics

Student’s *t*-test and two-way repeated measures analysis of variance (ANOVA) were performed using Excel Statistics (Statcel 2; Social Survey Research Information Co. Ltd., Tokyo, Japan). All values are presented as mean ± SEM. Values of *p* < 0.05 were considered statistically significant.

## Results

### SRRKO Mice Show Facilitated Long-Term Extinction Following Immediate Extinction Training

**Figure 1:**
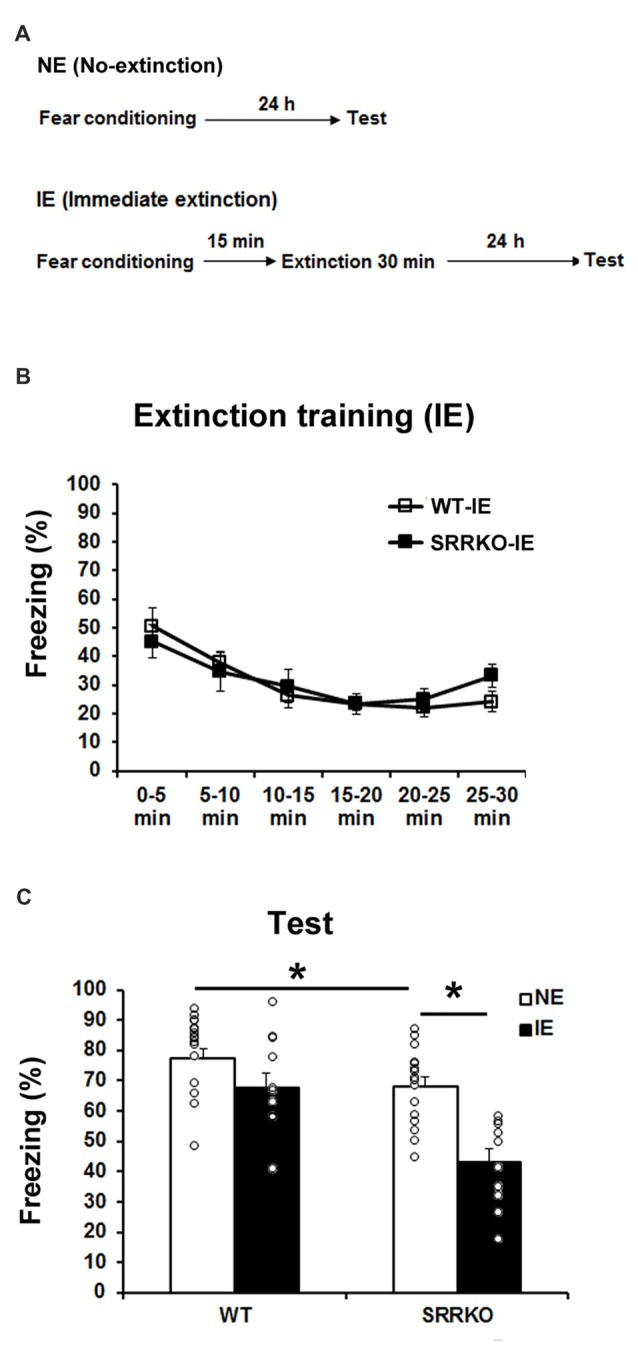
Immediate extinction (IE) of contextual fear memory is facilitated in SRRKO mice. **(A)** Experimental schedule for contextual fear conditioning, IE and the recall test. **(B)** During the extinction training, there was no significant difference in within-session extinction between SRRKO-IE (*n* = 10) and wild-type (WT)-IE (*n* = 11) mice. **(C)** During the recall test, SRRKO-IE mice exhibited significantly lower levels of freezing than did no-extinction (NE) group of SRRKO mice (SRRKO-NE, *n* = 16; *p < 0.05). There was no significant difference in freezing levels between WT-IE and WT-NE mice (WT-NE, *n* = 16). Data are presented as means ± SEM.

We first examined the effect of D-serine deficiency on extinction of contextual fear memory following IE training. WT (WT-IE) and SRRKO (SRRKO-IE) mice were subjected to a 30-min extinction session 15 min after fear conditioning and the extinction recall test was conducted 24 h after extinction training (Figure 1A). To evaluate the efficacy of IE training, another no-extinction (NE) group of WT and SRRKO mice was used for comparative analysis. There was no significant difference in the freezing level between WT and SRRKO mice during the first 5 min of the extinction session, indicating that short-term contextual fear memory was comparable between the two genotypes (Figure 1B). During the extinction training, two-way repeated measures ANOVA revealed a main effect of time (*F*_5,114_ = 8.36, *p* < 0.001). There was no main effects of genotype (*F*_1,114_ = 0.19, *p* = 0.66) and genotype × time interaction (*F*_5,114_ = 0.62, *p* = 0.69). During the recall test of extinction memory, two-way repeated measures ANOVA revealed main effects of extinction training (*F*_1,49_ = 20.14, *p* < 0.001), genotype (*F*_1,49_ = 17.63, *p* < 0.001), and genotype × extinction training interaction (*F*_1,49_ = 4.07, *p* = 0.049), suggesting a facilitated contextual fear extinction in SRRKO mice (Figure 1C). *Post hoc* analyses showed that SRRKO-IE mice exhibit significantly lower levels of freezing than did SRRKO-NE mice (Figure 1C, *p* < 0.05; Student’s *t* test). There was no significant difference in freezing levels between WT-IE and WT-NE mice. Notably, SRRKO-NE mice exhibited significantly lower levels of freezing than did WT-NE mice, suggesting that long-term contextual fear memory was impaired in SRRKO mice (Figure 1C, *p* < 0.05; Student’s *t* test).

### Long-Term Extinction Following Delayed Extinction Training Was Unchanged in SRRKO Mice

**Figure 2:**
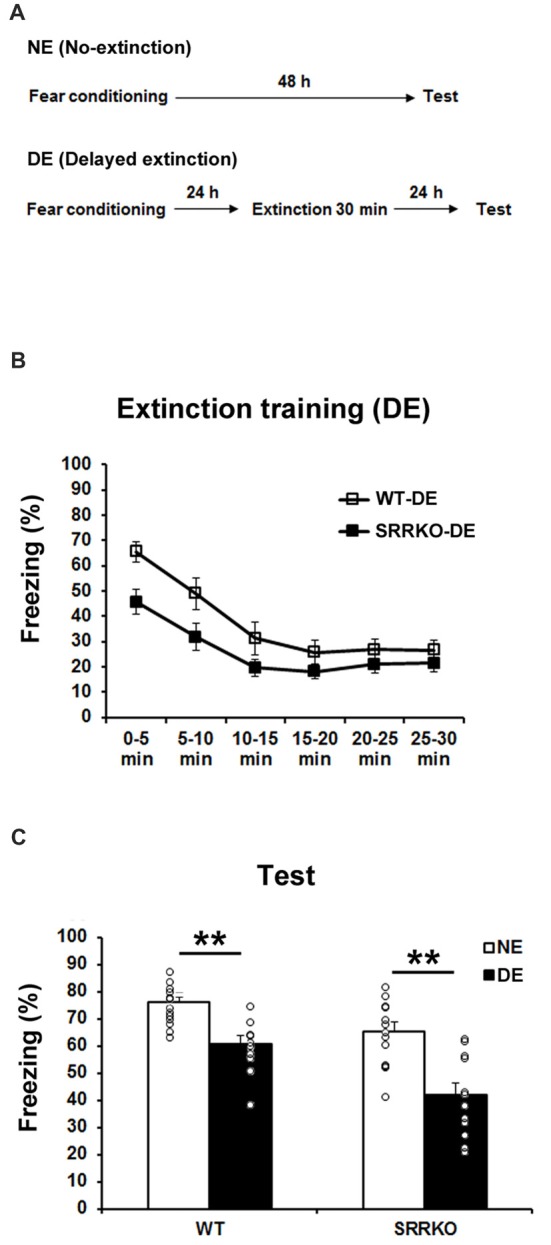
Delayed extinction (DE) of contextual fear memory is comparable between WT and SRRKO mice. **(A)** Experimental schedule for contextual fear conditioning, DE and the recall test. **(B)** During the extinction training, SRRKO-DE (*n* = 12) mice exhibited significantly lower levels of freezing than did WT-DE mice (*n* = 10). However, there was no significant difference in within-session extinction between SRRKO-DE and WT-DE mice. **(C)** During the recall test, WT-DE and SRRKO-DE mice exhibited significantly lower levels of freezing than did WT-NE and SRRKO-NE mice, respectively (WT-NE, *n* = 13; SRRKO-NE, *n* = 13; **p < 0.001). Data are presented as means ± SEM.

We next investigated the effect of D-serine deficiency on extinction of contextual fear memory following DE training. WT (WT-DE) and SRRKO (SRRKO-DE) mice were subjected to a 30-min extinction session 24 h after the fear conditioning and the extinction recall test was conducted 24 h after extinction training (Figure 2A). Another NE group of WT and SRRKO mice was used for comparative analysis. During the extinction training, two-way repeated measures ANOVA revealed main effects of time (*F*_5,120_ = 17.25, *p* < 0.001) and genotype (*F*_1,120_ = 18.85, *p* < 0.001). There was no main effect of genotype × time interaction (*F*_5,120_ = 0.91, *p* = 0.47; Figure 2B). During the recall test of extinction memory, two-way repeated measures of ANOVA revealed main effects of extinction training (*F*_1,44_ = 36.35, *p* < 0.001) and genotype (*F*_1,44_ = 17.97, *p* < 0.001) but not genotype × extinction training interaction (*F*_1,44_ = 1.47, *p* = 0.23; Figure 2C). *Post hoc* analyses showed that WT-DE and SRRKO-DE mice exhibited a significant reduction in freezing levels than did WT-NE and SRRKO-NE mice, respectively (WT-DE vs. WT-NE, *p* < 0.001; SRRKO-DE vs. SRRKO-NE, *p* < 0.001; Student’s *t* test).

### Post-retrieval Extinction Was Impaired in SRRKO Mice

**Figure 3:**
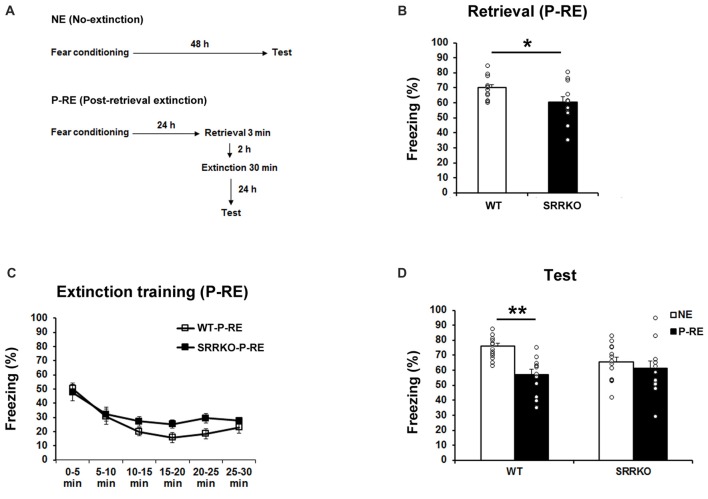
Post-retrieval extinction (P-RE) of contextual fear memory is impaired in SRRKO mice. **(A)** Experimental schedule for contextual fear conditioning, P-RE and the recall test. **(B)** During the retrieval session, SRRKO-P-RE mice (*n* = 12) exhibited significantly lower levels of freezing than did WT-P-RE mice (*n* = 12; *p < 0.05). **(C)** SRRKO-P-RE mice showed impaired within-session fear extinction during the P-RE training. **(D)** During the recall test, WT-P-RE mice exhibited significantly lower levels of freezing than did WT-NE mice (WT-NE, *n* = 13; **p < 0.001). The freezing levels in SRRKO-P-RE and SRRKO-NE (*n* = 13) mice were comparable. Data are presented as means ± SEM.

Upon retrieval, consolidated memory returns to a transient destabilized state and is prone to modification (Nader et al., 2000; Nader and Einarsson, 2010). To test the effect of D-serine deficiency on P-RE of contextual fear memory, WT (WT-PRE) and SRRKO (SRRKO-PRE) mice were exposed to a training context for 3 min and then subjected to a 30-min extinction session 2 h later (Figure 3A). The extinction recall test was conducted 24 h after extinction training. Another NE group of WT and SRRKO mice was used for comparative analysis. During the 3-min retrieval session, SRRKO mice showed a significantly lower level of freezing compared to WT mice (Figure 3B, *p* < 0.05, Student’s *t* test). During extinction training, two-way repeated measures ANOVA revealed main effects of time (*F*_5,132_ = 11.63, *p* < 0.001), genotype (*F*_1,132_ = 3.90, *p* = 0.05) and genotype × time interaction (*F*_5,132_ = 3.05, *p* = 0.01; Figure 3C). During the recall test of extinction memory, two-way repeated measures ANOVA revealed main effects of extinction training (*F*_1,46_ = 10.96, *p* = 0.002), and genotype × extinction training interaction (*F*_1,46_ = 4.35, *p* = 0.04; Figure 3D). There was no main effect of genotype (*F*_1,46_ = 0.93, *p* = 0.34). *Post hoc* analyses showed that WT-P-RE mice exhibit significantly lower levels of freezing than did WT-NE mice (*p* < 0.001; Student’s *t* test). In contrast, such an effect of P-RE training was not observed in SRRKO mice.

### SRRKO Mice Exhibit No Changes in Locomotor Activity and Anxiety-Like Behavior

**Figure 4:**
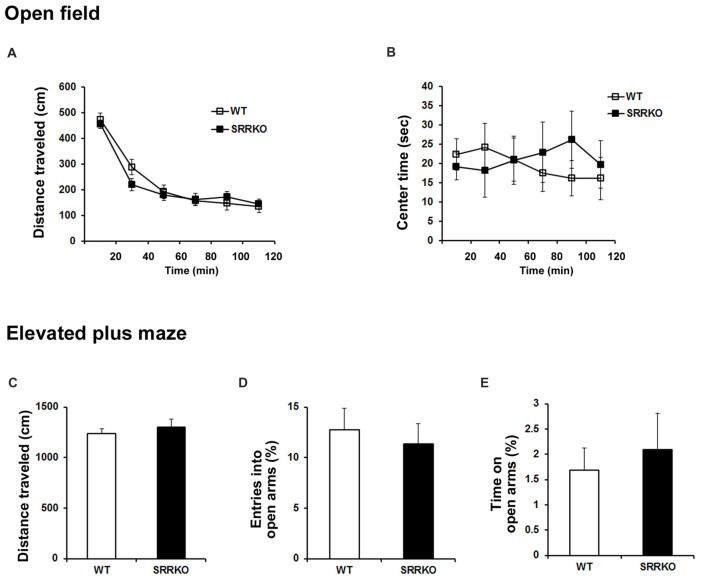
SRRKO mice exhibit no changes in locomotor activity and anxiety level compared with WT mice. **(A,B)** In the open field test, the distance of travel throughout the apparatus **(A)** and the time spent in the center zone of the open field **(B)** were comparable between WT and SRRKO mice (*n* = 20). **(C–E)** In the elevated plus maze test, there were no significant differences in the distance of travel throughout the apparatus **(C)**, the percentage of entries into **(D)** and the time spent in the open arms **(E)** between the two genotypes. Data are presented as mean ± SEM.

We examined the locomotor activity and anxiety-like behavior in the open field and the elevated plus maze tests. In the open field test, the distance of travel throughout the apparatus (Figure 4A, *F*_1,228_ = 0.51, *p* = 0.48; two-way repeated measures ANOVA) and the time spend in the center zone of the open field (Figure 4B, *F*_1,228_ = 0.225, *p* = 0.64; two-way repeated measures ANOVA) were comparable between WT and SRRKO mice. In the elevated plus maze test, the distance of travel throughout the apparatus (Figure 4C, *p* = 0.49; Student’s *t* test), the percentage of entries into (Figure 4D, *p* = 0.63; Student’s *t* test) and the time spend in the open arms (Figure 4E, *p* = 0.63; Student’s *t* test) did not differ between the two genotypes.

### Expression Level of AMPAR GluA1 Subunit in the Synaptic Membrane Fraction Is Higher in SRRKO Than WT Mice After Post-retrieval Extinction Training

**Figure 5:**
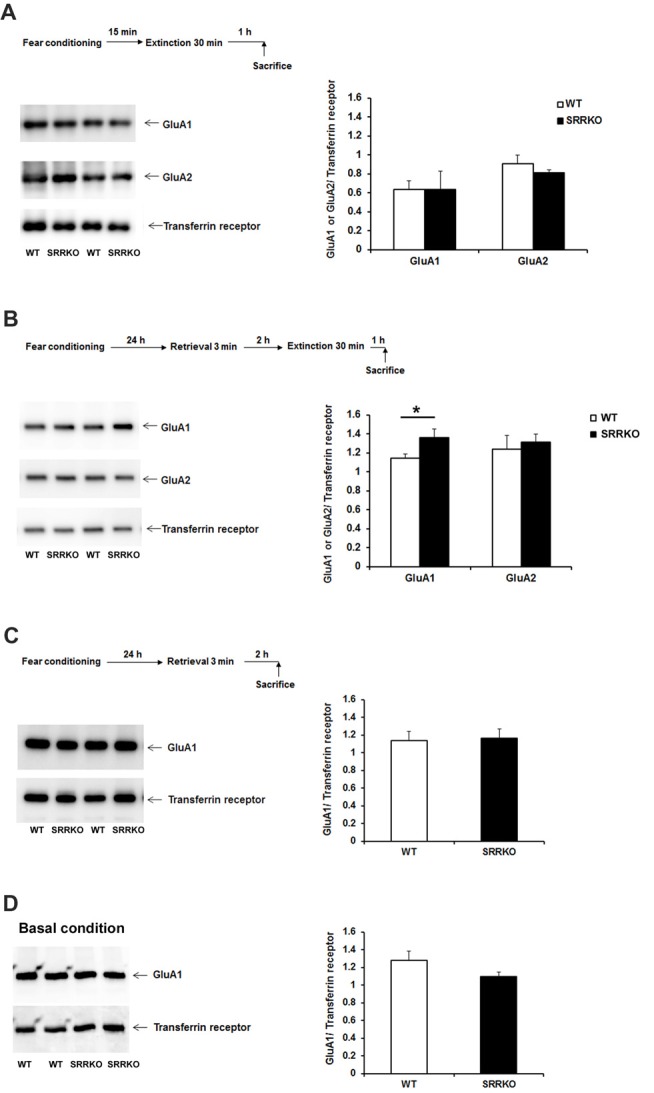
SRRKO mice express higher levels of α-amino-3-hydroxy-5-methyl-4-isoxazolepropionic acid-type glutamate receptor (AMPAR) GluA1 subunit in the synaptic membrane than do WT mice after P-RE. **(A)** Expressions of AMPAR GluA1 and GluA2 subunit in the hippocampal synaptic membrane fraction 1 h after IE were comparable between WT (*n* = 3) and SRRKO (*n* = 3) mice. **(B)** Expressions of the GluA1 and GluA2 subunit in the hippocampal synaptic membrane fraction 1 h after P-RE. SRRKO mice exhibited significantly higher levels of GluA1 subunit in the synaptic membrane fraction than did WT mice (*n* = 4, *p < 0.05). **(C,D)** WT and SRRKO mice showed similar levels of GluA1 in the synaptic membrane fraction of the hippocampus 2 h after retrieval **(C)** or under basal conditions **(D)**. Data are presented as means ± SEM.

We next investigated what molecular mechanism is involved in the differential behavioral effect of immediate and P-RE training in WT and SRRKO mice. Endocytosis of AMPAR and the resulting synaptic depotentiation are indispensable molecular mechanisms for the behavioral effect of extinction (Dalton et al., 2008; Rao-Ruiz et al., 2011). In the adult hippocampus, AMPAR consists mainly of GluA1/GluA2 hetero-tetramers (Shi et al., 2001). Therefore, we examined the levels of GluA1 and GluA2 subunits in the synaptic membrane fraction of the hippocampus 1 h after immediate or P-RE training by western blot analysis. The expression of GluA1 and GluA2 was normalized to the transferrin receptor, a plasma membrane protein that was not expected to change following long-term depression (LTD)-inducing treatment (Davies et al., 2008). There was no significant difference in expression of AMPAR GluA1 and GluA2 subunit in synaptic membrane fraction of hippocampus 1 h after IE training (Figure 5A). The expression of AMPAR GluA1 subunit in the synaptic membrane fraction of hippocampus was significantly higher in SRRKO mice than in WT mice 1 h after P-RE training (Figure 5B, *p* = 0.032, Student’s *t* test). The level of GluA2 in the membrane fraction was similar between two genotypes after P-RE. We then examined whether the level of GluA1 in the synaptic membrane fraction was different between WT and SRRKO mice 2 h after retrieval (Figure 5C) or in the naïve mice (Figure 5D). At these two conditions, WT and SRRKO mice showed similar levels of GluA1 in the hippocampal synaptic membrane fraction.

### Administration of D-Serine Rescues Post-retrieval Extinction Deficit in SRRKO Mice

**Figure 6:**
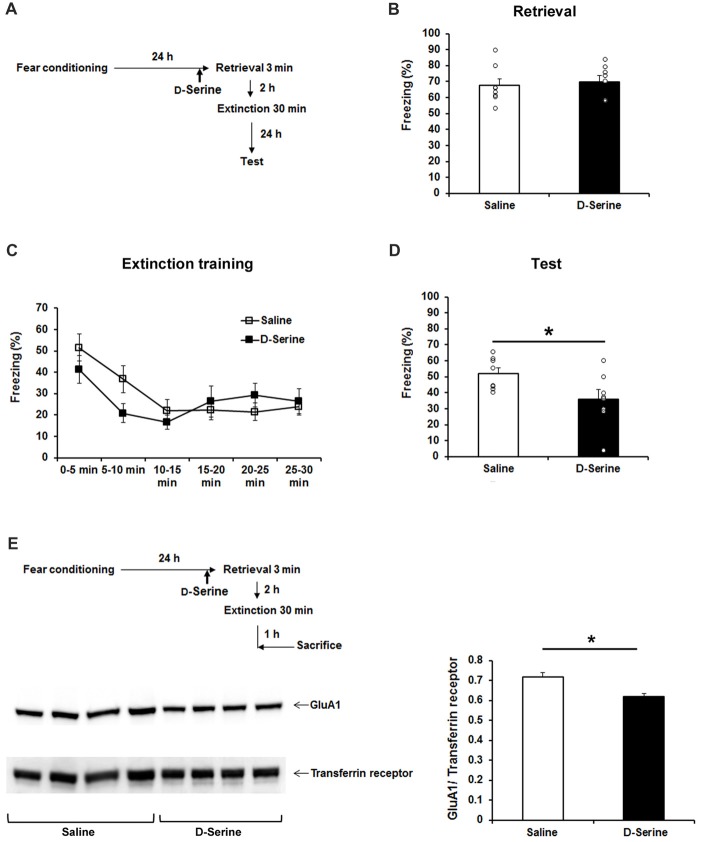
D-Serine treatment rescues the deficit of P-RE in the SRRKO mice. **(A)** Experimental schedule for the behavioral rescue experiment. **(B)** D-Serine-treated SRRKO mice (*n* = 8, D-Serine) exhibited similar levels of freezing compared with saline-treated SRRKO mice (*n* = 8, Saline) during the 3-min retrieval session. **(C)** During the extinction training conducted 2 h after retrieval, there was no significant difference in within-session extinction between D-serine- and saline-treated SRRKO mice. **(D)** During the recall test of extinction memory, D-serine-treated SRRKO mice exhibited significantly lower levels of freezing than did the saline-treated SRRKO mice (*p < 0.05). **(E)** Western blot analysis revealed a significantly lower level of GluA1 in the synaptic membrane fraction of D-serine-treated SRRKO mice (*n* = 4) than in saline-treated SRRKO mice (*n* = 4) 1 h after P-RE (*p < 0.05). Data are presented as means ± SEM.

To confirm that impaired P-RE in SRRKO mice is specifically attributed to reduced synthesis of D-serine by SRR disruption, we performed a rescue experiment by administrating D-serine 10 min before retrieval session (Figure 6A). The dose of D-serine (2.7 g/kg) was chosen based on the results of previous behavioral studies (Kanahara et al., 2008; Matsuda et al., 2010). The freezing level during the 3-min retrieval (Figure 6B, *p* = 0.67, Student’s *t* test) and extinction training (Figure 6C, *F*_1,84_ = 0.86, *p* = 0.36, two-way repeated measures ANOVA) was comparable between saline- and D-serine-treated SRRKO mice. However, during the recall test of extinction memory, the D-serine-treated SRRKO mice showed significantly lower levels of freezing than did the saline-treated SRRKO mice (Figure 6D, *p* = 0.036, Student’s *t* test). We then examined whether administration of D-serine can reduce expression of GluA1 in the hippocampal synaptic membrane fraction after P-RE training (Figure 6E). D-Serine-treated SRRKO mice showed significantly lower levels of GluA1 in the hippocampal synaptic membrane fraction than did saline-treated SRRKO mice 1 h after P-RE (*p* = 0.013, Student’s *t* test).

## Discussion

Increasing evidence shows that pharmacological manipulation of glutamatergic neurotransmission mediated at the NMDAR glycine-binding site may provide an innovative treatment approach for anxiety disorders, such as PTSD. In the present study, we found that SRR disruption impairs P-RE but not DE and in contrary, facilitates IE of contextual fear memory. These results suggest that the action of D-serine differentially regulates extinction of contextual fear memory, depending on the length of time between the initial learning and the extinction session.

Newly acquired memory is progressively made permanent through a process of consolidation (Dudai, 2004). Attention has been paid to behavioral interventions given soon after fear conditioning, with aim to disrupt long-term fear memory by impeding the consolidation process. However, in our examination, such an IE procedure failed to reduce expression of contextual fear in WT mice, which is in agreement with several previous studies where an IE after contextual and auditory fear conditioning showed null effect in extinguishing original fear memory (Maren and Chang, 2006; Schiller et al., 2008; Woods and Bouton, 2008; Archbold et al., 2010). The notable finding in this work is that IE intervention significantly reduced contextual fear in the SRRKO mice. It has been proposed that memory traces of both conditioning and extinction persist after extinction training, and that performance during the subsequent recall test is governed by the extent to which each process is weighted. Both consolidation and extinction of fear memory require the activation of the NMDAR (Abel and Lattal, 2001; Santini et al., 2001). SRRKO mice exhibited impaired long-term contextual fear memory (Figure 1C, WT-NE vs. SRRKO-NE) but normal short-term memory as shown by the freezing levels within the first 5 min of IE training (Figure 1B). Together with the finding that D-serine treatment 10 min before retrieval test failed to alter freezing levels in SRRKO mice (Figure 6B), these results suggest that D-serine is required for the NMDAR-mediated consolidation of a newly acquired fear memory but not long-term memory retrieval. Consistent with this finding, a previous report has demonstrated that D-cycloserine facilitates consolidation of fear (Handford et al., 2014). Together, it is possible that D-serine is preferentially involved in the consolidation of an acquired fear memory rather than extinguishing the original memory when the behavioral intervention is performed soon after learning.

Upon retrieval, consolidated memory returns to a transient destabilized state and is prone to modification, offering a potential target period during which the aberrant memories underlying psychiatric disorders can be erased (Nader et al., 2000; Nader and Einarsson, 2010). Recent findings suggest that delivery of extinction shortly after memory retrieval might erase consolidated memories (Monfils et al., 2009; Schiller et al., 2010). Memory destabilization is a prerequisite for the beneficial effects of the P-RE (Pineyro et al., 2014). Previous works have indicated that NMDAR antagonists prevent fear memories from becoming labile after a reminder (Ben Mamou et al., 2006; Milton et al., 2013). Therefore, it is conceivable that the action of D-serine on the NMDAR glycine-binding site contributes to destabilization of reactivated memory, and thereby enhances the efficiency of extinction of fear memory. Supporting this hypothesis, administration of D-serine before a retrieval session rescued impaired P-RE of contextual fear memory in SRRKO mice.

Several recent studies have suggested that endocytosis of AMPAR is necessary for memory remodeling after retrieval (Clem and Huganir, 2010). Presenting a conditioned stimulus 1 h after retrieval session resulted in dephosphorylation of the AMPAR GluA1 subunit at Ser^845^, which is usually followed by the endocytosis of AMPAR (Monfils et al., 2009). Furthermore, it was recently reported that retrieval of contextual fear memory leads to the removal of the AMPAR GluA1 and GluA2 subunits from the hippocampal synaptic membrane and that blocking endocytosis of GluA2-containing AMPAR in the hippocampus 1 h before P-RE prevents long-term attenuation of the expression of contextual fear memory (Rao-Ruiz et al., 2011). Our western blot analysis revealed significantly higher expression of AMPAR GluA1 but not GluA2 subunit in the hippocampal synaptic membrane fraction of SRRKO mice than in WT mice after P-RE. In the rescue experiment, administration of D-serine into SRRKO mice reduced the expression of GluA1 in the synaptic membrane fraction after P-RE. NMDAR activation controls AMPAR endocytosis during hippocampal LTD (Unoki et al., 2012). Therefore, the action of D-serine on the NMDAR glycine-binding site may facilitate the endocytosis of GluA1-containing AMPAR after P-RE, which contributes to memory destabilization and allows long-term attenuation of contextual fear memory. Unlike P-RE, IE training did not exert effect on the expression of AMPAR subunits in synaptic membrane fraction of hippocampus, which is not conflict with the mentioned concept that D-serine is preferentially involved in the consolidation of an acquired fear memory rather than extinguishing the original memory.

A notable finding in the present study is that D-serine plays opposite role in IE and P-RE of contextual fear memory. Although reconsolidation and consolidation processes share many similar cellular and molecular characteristics, there are, in fact, some differences in the molecules required for the two memory processes (Lee, 2010). It is also possible that different neural circuits may mediate fear extinction during the consolidation and reconsolidation phase. Using a context retrieval-dependent memory-enhancement model in rats, Ye et al. (2017) demonstrated that direct dorsal hippocampal–prelimbic cortex connections strengthen memory through reconsolidation while suppressing extinction. Recently, it is proposed that D-serine levels can be regulated in distinct brain regions as a function of their involvement in a specific behavioral task (Papouin et al., 2017). Therefore, we speculate that D-serine availability is dynamically regulated in brain regions such as the hippocampus and prefrontal cortex during the course of memory processing that leads to the differential involvement of D-serine in IE, DE and P-RE of fear memory. Overall, our findings suggest that combining drugs enhancing the NMDAR function with P-RE may yield optimal outcomes for the treatment of fear-related disorders such as PTSD.

## Author Contributions

RI, TM and HM designed the studies. RI, KT and HM wrote the manuscript. RI, KT and GT performed the experiments and analyzed data.

## Conflict of Interest Statement

The authors declare that the research was conducted in the absence of any commercial or financial relationships that could be construed as a potential conflict of interest.

## Footnotes

^1^http://www.mouse-phenotype.org/
